# Geneticization in the genomic era: a scoping review of ethical, clinical, and sociocultural transformations

**DOI:** 10.3389/fsoc.2025.1675678

**Published:** 2026-01-05

**Authors:** Safa Shaheen, Mohammed Ghaly

**Affiliations:** 1 College of Health and Life Sciences, Hamad bin Khalifa University, Education City, Doha, Qatar; 2 College of Islamic Studies, Hamad bin Khalifa University, Education City, Doha, Qatar

**Keywords:** geneticization, Lippman, ELSI, biological citizenship, biosociality, bioethics

## Abstract

Geneticization is a concept originally introduced by Abby Lippman to critique the growing dominance of genetic explanations in health, identity, and society. Over the decades, the notion of geneticization has undergone significant development across various academic fields including sociology, bioethics, clinical medicine, and cultural studies, highlighting its broad relevance and impact on multiple areas of research. We conducted a scoping review of 25 peer-reviewed studies from 2011 and 2024, to investigate how the concept has been taken up, redefined, and challenged across multiple disciplines. Guided by two central research questions: (1) What are the prevailing themes surrounding geneticization in recent scholarship? and (2) To what extent do Lippman’s original concerns remain relevant? the review synthesizes insights from these studies, categorizing them across sociological, clinical, and ethical dimensions. Findings reveal a shift from deterministic framings toward more complex understandings, such as enlightened geneticization, biosociality, and biological citizenship, which highlight individuals’ agency in interpreting genetic information. At the same time, the review identifies ongoing risks of genetic reductionism in areas such as race, identity, reproduction, and education. The results underscore that while the term “geneticization” has evolved in both use and meaning, it remains a critical analytical lens for evaluating the ethical, legal, and social implications (ELSI) of genetic technologies. The review concludes by emphasizing the continued relevance of interdisciplinary inquiry and ethical vigilance in the genomic era.

## 1 Introduction

The term geneticization was first articulated in the early 1990s by Canadian epidemiologist and women’s health scholar (Lippman, 1991, 1992). In a series of influential publications, Lippman (1998) and Lippman (1993) offered a critical appraisal of the growing incorporation of genetic technologies into routine healthcare, with particular concern for their implications in prenatal screening and women’s reproductive health. Lippman (1991) warned that this trend risked reinforcing genetic determinism and reductionism, whereby individual identity and health outcomes were interpreted primarily through the lens of genetic composition, potentially silencing alternative, non-genetic explanations for disease and well-being. Her analysis laid the groundwork for a broader debate on how genetics shapes not only scientific practice but also cultural understandings of selfhood, responsibility, and belonging. Subsequent scholarships expanded Lippman’s framework, linking geneticization to broader sociological and ethical debates about the medicalization of everyday life (Gerhardt, 1989; Clarke et al., 2003). Like medicalization and biomedicalization, geneticization denotes the extension of biomedical reasoning into new domains, but with a distinctive focus on the authority of genetic knowledge.

The origins and applications of the concept of geneticization have been traced across a range of historical and disciplinary contexts. Flitner (2003) links the early use of genetics to state-led agricultural modernization programs in the 1920 and 1930s, where genetic science was intertwined with social Darwinist ideologies, reflecting the politicization of heredity in shaping populations. In the realm of medicine, Hoedemaekers and ten Have (1998) have critically examined the influence of geneticization on preventive screening and counseling practices, emphasizing its role in reshaping clinical paradigms. Ten Have (2001) further argues that Western societies are undergoing a profound redefinition of personhood, where individuals are increasingly interpreted through the lens of DNA codes and genetic language. He proposes that geneticization functions as a heuristic tool to redirect bioethical debates toward the interpersonal, institutional, and cultural dimensions of emerging genetic knowledge. Similarly, Stempsey (2006) characterizes geneticization as a transformative force in both medical diagnostics and societal understanding, while Horstman (2008) warns that this shift may marginalize social and cultural explanations of health and illness, privileging biological determinism over more holistic frameworks.

Building on earlier critiques, Hedgecoe (2009) offers an important empirical contribution by emphasizing that geneticization is not a uniform or simplistic process, but one shaped by complex individual and social interpretations of genetic information. His work underscores the ethical stakes of geneticization, arguing that it fosters a neo-ontological framing of disease where genetic identity becomes foundational to one’s being and may inadvertently promote genetic reductionism, with potential consequences such as discrimination and social inequality (Hedgecoe, 2009).

Over time, the concept has evolved beyond its original critique of reductionism to encompass more complex and context-sensitive analyses of how genetics intersects with identity, kinship, policy, and governance. As genetic technologies are increasingly embedded within healthcare systems globally, the demand for a multifaceted and interdisciplinary analysis of this phenomenon has become more pronounced. Scholarly engagement with geneticization has spanned various domains, which can be broadly categorized into three principal areas of investigation:

*Sociology, anthropology, and culture:* where geneticization is analyzed in relation to social structures, identity formation, and cultural narratives surrounding health and disease.

*Ethics and bioethics:* focusing on the moral, philosophical, and policy-related implications of genetic technologies, particularly concerning autonomy, consent, and equity.

*Clinical practice and medicine:* exploring the impact of geneticization on medical decision-making, patient care, and the role of genetic information in diagnostics and treatment.

This interdisciplinary perspective highlights the importance of moving beyond a narrowly biomedical interpretation of geneticization, urging a more comprehensive examination of its social, ethical, and clinical ramifications.

The rise of emerging medical paradigms such as personalized medicine, which is predicated on an individual’s genomic profile, has intensified the integration of genetic information into contemporary healthcare strategies. A key aim of global large-scale genome initiatives is not only to advance the development of precision and personalized medicine, but also to define the boundaries between normal and pathological genomic variation (Kovanda et al., 2021). These efforts are fundamentally informed by insights generated through genomic research. Moreover, the expanding societal reach of genetics is evident in the adoption of legal and policy frameworks in certain countries that require genetic testing as a prerequisite for marriage, underscoring the deepening influence of genomics in both medical and socio-legal domains, particularly over the past decade (Bener et al., 2019).

Despite a rich body of theoretical and empirical work, two comprehensive reviews—by Arribas-Ayllon (2016) and Weiner et al. (2017) have largely defined the state of the field to date. Both demonstrated how the term had shifted from a critical warning about determinism to a heuristic tool for studying the social and epistemic consequences of genomic science. However, these reviews were limited by their temporal scope and disciplinary focus. Arribas-Ayllon’s work centered on biosociality and disease classification, while Weiner and colleagues emphasized theoretical debates within sociology and science and technology studies (STS). The present review builds on these foundations by systematically synthesizing literature published between 2011 and 2024, a period marked by major genomic, technological, and ethical transformations. It integrates findings across sociology, bioethics, and clinical medicine—disciplines rarely examined together, thereby providing a broader interdisciplinary assessment of how the notion of geneticization has evolved in the postgenomic era.

In addition to this disciplinary synthesis, the present study situates geneticization within current public discourse, where the social and ethical stakes of genetic knowledge have become increasingly visible. The rise of direct-to-consumer (DTC) genetic testing has enabled individuals to access and interpret their genomic data outside clinical contexts, reshaping notions of ancestry, health, and identity (Friedman and Anderson, 2024; Majumder et al., 2021). Artificial intelligence (AI) applications in genomics ranging from predictive diagnostics to algorithmic risk profiling have further blurred the boundary between medical science and data capitalism, raising new concerns about transparency, bias, and data sovereignty (Dara et al., 2025; Ahmed et al., 2023; Dias and Torkamani, 2019). Similarly, the use of genetic testing in forensics and immigration governance where ethical dilemmas around consent, unintended findings, and database use are increasingly prominent, illustrate how geneticization extends beyond healthcare into legal, investigative and political arenas (Sessa et al., 2024; Barata et al., 2015; Duster, 2015). Meanwhile, advances in epigenetics complicate earlier deterministic framings by emphasizing the reciprocal influence of environment and social experience on gene expression (Meloni, 2015; Lock, 2015). Together, these developments underscore that the discourse on geneticization remains both empirically and normatively vital, requiring renewed attention to its ethical, social, and policy implications in a rapidly evolving genomic landscape.

Accordingly, this review examines how recent scholarship has reinterpreted and operationalized the concept of geneticization across diverse contexts. It asks: (1) What are the prevailing themes and trajectories in contemporary debates on geneticization? and (2) To what extent do Lippman’s original concerns remain relevant in the era of precision medicine, big data, and global genomics? By synthesizing insights from 25 recent studies, the review seeks to advance an updated, interdisciplinary understanding of geneticization as both a social process and an analytical framework for evaluating the ethical, legal, and social implications (ELSI) of modern genetics.

## 2 Materials and methods

**Figure 1:**
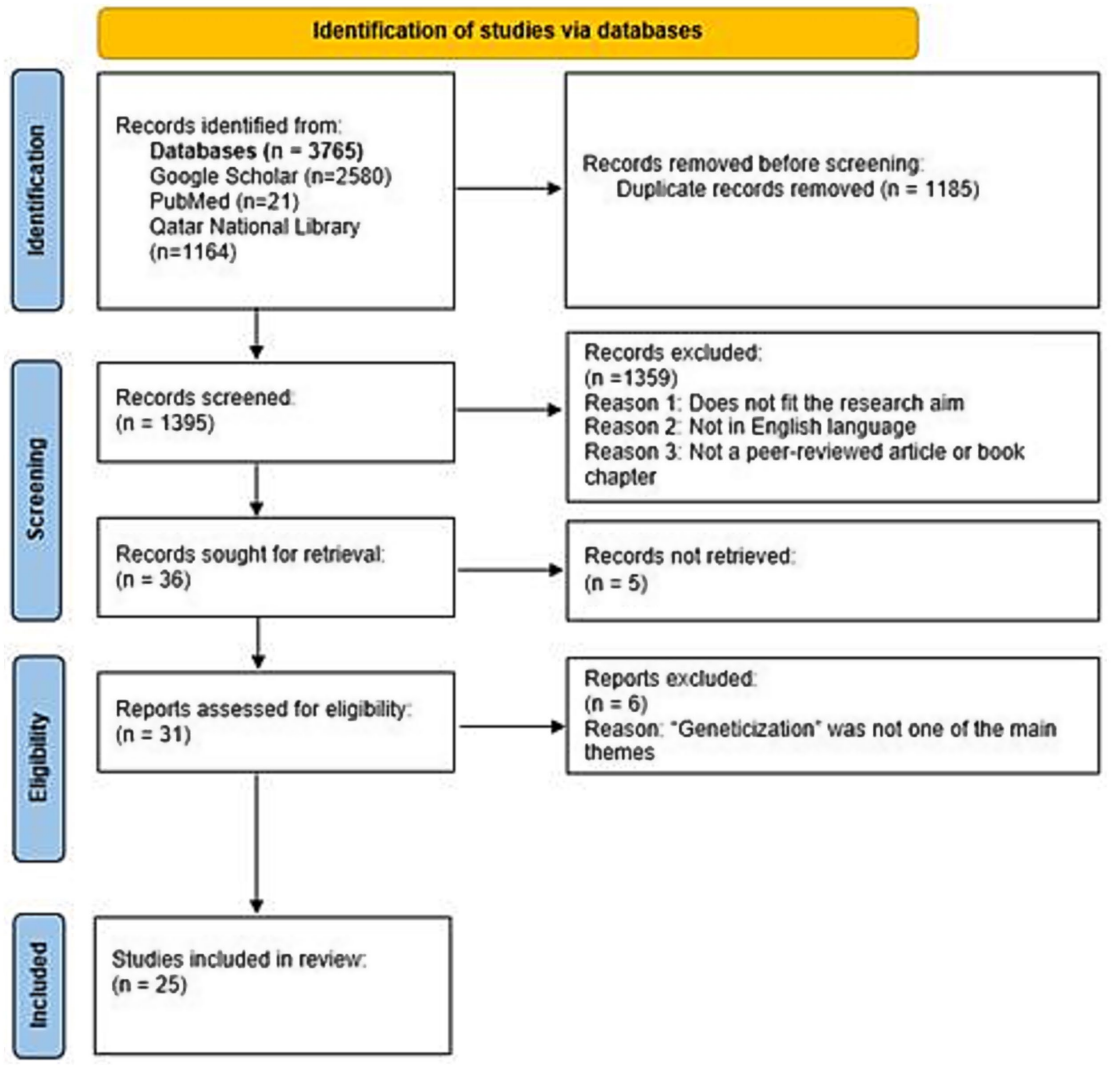
Geneticization literature review flow diagram (framework adapted from PRISMA 2020 flow diagram adapted for scoping review).

This study followed a scoping review design to map how the concept of geneticization has been defined, applied, and contested across sociology, bioethics, clinical medicine, and related fields. PRISMA-ScR guidelines were followed (Tricco et al., 2018) to ensure transparency, replicability, and rigor in the identification, screening, and selection of studies. A scoping review was selected because the literature on geneticization is conceptually diverse, spans multiple disciplinary traditions, and includes heterogeneous methods. The study selection process is presented using a PRISMA 2020 flow diagram adapted for scoping review purposes (Figure 1). In this review, the term “records” is used to refer to all initial sources identified during the search process, encompassing journal articles, book chapters, and other types of publications. As the review progressed, the term “studies” was applied more specifically to those peer-reviewed records that were screened, assessed for eligibility, and ultimately included in the review.

### 2.1 Search strategy

An in-depth and systematic search was carried out across several electronic databases, including PubMed, Google Scholar, and digital collections accessible through the Qatar National Library. The search aimed to capture all relevant literature from January 2011 to December 2024. The terms used in the search strategy included the keyword variants: “geneticization” OR “geneticisation,” as both spellings are used interchangeably in the literature. These search terms were applied to the titles, abstracts, and keywords of articles to ensure comprehensive coverage. The search terms were used consistently across all databases utilized for the study.

### 2.2 Inclusion and exclusion criteria

To maintain a focused scope, studies were included based on the following inclusion criteria:

- Articles published between 2011 and 2024.
- Peer-reviewed publications in English.
- Studies in which “geneticization” or “geneticisation” formed the central analytical or conceptual theme.

Studies were excluded if:

- The term “geneticization” was only mentioned briefly or in passing.
- The article did not engage with the theoretical, ethical, or social dimensions of geneticization in a substantive manner.
- Full text was unavailable.

Because scoping reviews aim to map research rather than restrict it, no limitations on study design or methodology were imposed.

### 2.3 Screening and selection process

The initial search resulted in the generation of a total of 3,765 articles from the different databases such as Google Scholar (*n* = 2,580), PubMed (*n* = 21) and databases (around 200) accessible through Qatar National Library (*n* = 1,164). Following the removal of duplicate records (*n* = 1,185), a total of 1,395 records remained. These were screened for relevance to the research focus. A total of 1,359 records were excluded based on the following criteria: not in English language, not a peer-reviewed article or edited book chapter, or insufficient alignment with the core research questions. After this, 36 records remained which were searched for full text availability. Five full-text records could not be retrieved. The remaining 31 records—comprising 27 peer-reviewed journal articles and 4 book chapters, met the inclusion criteria and were assessed in detail for their contributions to the study’s focus on the concept of geneticization within the specified time period (see Figure 1). The relatively low number of results retrieved from PubMed likely reflects the disciplinary distribution of scholarship on geneticization. Since geneticization is a concept originating primarily within sociology, anthropology, STS, and bioethics, much of the relevant scholarship is published in journals outside the biomedical domain. As PubMed predominantly indexes biomedical and physical science literature, it returned comparatively fewer records than other databases. Furthermore, geneticization is not represented in PubMed’s controlled vocabulary—Medical Subject Headings (MeSH), which limits the sensitivity of keyword-based searches for conceptual or theoretical work. In contrast, Google Scholar and QNL databases employ broader full-text indexing and include social science journals, book chapters, and gray literature, resulting in a substantially higher number of retrieved sources. These disciplinary and indexing differences were taken into account when interpreting database yields.

Bibliographic references of selected studies were scanned to identify any additional relevant literature, but this did not result in any additions. Screening of titles and abstracts was conducted by the first author, while the second author reviewed and discussed uncertain or borderline cases. Any uncertainties or disagreements regarding the inclusion of studies were addressed through discussion between the two authors until consensus was reached; no third reviewer was involved at any stage of the screening or selection process. Microsoft Excel was used for record tracking, inclusion/exclusion decisions, and data visualization.

### 2.4 Data extraction and analysis

A detailed content analysis was then conducted. All 31 records were read multiple times, and a standardized data extraction table was developed by the first author to systematically capture key elements of each study, including research objectives, methodological approach, outcomes of interest, and other relevant details (see Table 1). This table facilitated structured comparison and synthesis across studies. Subsequently, backward citation tracking was employed, whereby the reference lists of the already included studies were systematically examined to identify additional relevant literature, but this did not result in the inclusion of new studies within the specified time frame. The second author reviewed and provided feedback on the data collection procedures, analytical process, and preliminary findings to enhance the reliability of the review. No formal risk-of-bias assessment was conducted, as scoping reviews aim to map the landscape of existing research rather than assess study quality.

**Table 1:** Representation of data extraction form.

Authors	Year	Type of record	Title	Research objectives	Methodology	Outcomes of interest
Tsekeris and Alexias	2012	Journal article	Science, genetic knowledge and the human body	To overview the intersection of geneticization and genetic counseling	Comprehensive critique of contemporary theoretical literature	Promotes critical thinking about being human and coping with genetic and bodily knowledge
Ankeny, RA	2017	Journal article	Geneticization in MIM/OMIM^®^?	Explores the evolution of MIM/OMIM^®^	Philosophical and historical analysis of MIM/OMIM^®^	The phenomenon of geneticization is not solely recent or always associated with genomic sequencing
Matthews, LJ	2024	Journal article	The geneticization of education and its bioethical implications	Examine the geneticization of education	Comprehensive critique of using Direct to Consumer (DTC) genetic testing for determining educational outcomes.	Explore both real and potential downstream bioethical implications and proposals for mitigating negative impacts

During the course of the content analysis, six records—5 journal articles and 1 book chapter, were excluded upon closer inspection since the concept of “geneticization” was not one of the main themes of the article or chapter—which is one of main criteria for inclusion. Although it is inherently challenging to guarantee that all relevant studies have been captured, the systematic approach adopted including iterative searching and citation tracking supports the conclusion that the review is sufficiently comprehensive and methodologically robust. We further categorized the studies into the 3 domains mentioned earlier (see Table 2 for included studies).

**Table 2:** Studies included in the literature review.

Category	Reference	Year	Title	Relevance/justification for inclusion
Sociology, anthropology and culture	Tsekeris and Alexias (2012)	2012	Science, genetic knowledge, and the human body	Overviewed geneticization and its relevance to genetic counseling
	Franklin (2013)	2013	From blood to genes?	Discussed consanguinity in the context of geneticization
	Arribas-Ayllon (2016)	2016	After geneticization	Reviewed and reconstructed the geneticization concept
	Hyun (2017)	2017	Geneticizing ethnicity and diet	Argued against geneticization of ethnicity and dietary habits in the context of anti-doping research
	Weiner et al. (2017)	2017	Have we seen the geneticisation of society?	Reviewed the literature on geneticization
	Dingel et al. (2019)	2019	“Why did I get that part of you?”	Studied geneticization of addiction as described by individuals in addiction treatment programs
	Strand and Källén (2021)	2021	I am a Viking!	Analyzed the construction of geneticized identity through the use of GATs
	Hunt and Merolla (2022)	2022	Genes and race in the era of genetic ancestry testing	Criticized the use of GAT for “the social deconstruction of whiteness” and thereby the geneticization of race
	Jacoby (2022)	2022	Commercially geneticizing race, ethnicity, and nation	Described the geneticization of race, ethnicity and identity through commercialized genetic tests
	Peters (2023)	2023	Racial-genomic interest convergence and the geneticization of Black families	Studied the geneticization of race
	Mannette (2021)	2021	Navigating a world of genes	Analyzed the concept of geneticization
	Shmidt and Donohue (2024)	2024	Invincible racism?	Criticized the geneticization of minoritized groups
Ethics and bioethics	Domaradzki (2019)	2019	Geneticization and biobanking	Described the geneticization of identity through biobanking and related ethical aspects
	Sharon (2014)	2014	New modes of ethical selfhood	Critiqued the geneticization thesis
	Roberts (2015)	2015	“good soldiers are made, not born”	Argued against geneticization of military ability and discussed the bioethical implications of the same
	Leźnicki (2020)	2020	Bioethical aspects of human geneticization	Discussed genetic enhancements and associated bioethical aspects
	Matthews (2024)	2024	The geneticization of education and its bioethical implications	Explained the geneticization of education and its bioethical implications
Clinics and medicine	Pavone and Arias (2012)	2012	Beyond the geneticization thesis	Critiqued the geneticization of PGD/PGS in the context of Spain
	Löwy (2014)	2014	How genetics came to the unborn	Described the geneticization of PND and in turn the geneticization of the unborn
	Navon and Eyal (2016)	2016	Looping genomes	Described the process of geneticization of autism
	Dekeuwer (2017)	(2017)	Conceptualization of genetic disease	Discussed the problem of geneticizing diseases
	Ankeny (2017)	2017	Geneticization in MIM/OMIM^®^?	Geneticization in Mendelian Inheritance cataloging
	Shim and Kim (2020)	2020	Contextualizing geneticization and medical pluralism	Situated the concepts of geneticization and medical pluralism within specific social, cultural, and institutional contexts to understand how they interact
	Alon et al. 2021	2021	Regulating reproductive genetic services	Discussed the geneticization of human reproduction
	Löwy (2022)	2022	Precision medicine	Discussed the geneticization of clinics

## 3 Results

### 3.1 Evaluation of the geneticization concept

The theoretical foundation of geneticization lies at the intersection of genetics and social theory, particularly in its critique of the reductionist and deterministic assumptions often embedded in genetic discourse (Arribas-Ayllon, 2016). Lippman’s original thesis framed geneticization as a set of processes through which genetic explanations increasingly displace social, environmental, and structural determinants in the understanding of health and disease (Weiner et al., 2017). This trend has contributed to the emergence of what some scholars characterize as a dominant discourse of genetic determinism, wherein genetic traits are positioned as the primary factors shaping individual identity, behavior, and health outcomes (Arribas-Ayllon, 2016; Weiner et al., 2017). However, recent scholarship has complicated this narrative, suggesting that Lippman’s initial formulation may overstate the uniformity and dominance of genetic discourse. Rather than treating genetic knowledge as a monolithic force, these scholars emphasize the situated and context-dependent ways in which individuals interpret and engage with genetic information. They argue that lay understandings of genetics are often mediated by personal experience, cultural values, and social context, resulting in a plurality of meanings and applications. The following sections explore some of the key extensions and reinterpretations of the geneticization thesis that have emerged in recent years.

#### 3.1.1 Enlightened geneticization

The concept of enlightened geneticization, introduced by Hedgecoe (2001b), represents a more nuanced iteration of geneticization—what he terms a “reasonable, non-extremist” position. This framing acknowledges the relevance of non-genetic factors but continues to prioritize genetic explanations as the primary causal mechanisms in understanding disease. Through a series of empirical investigations, Hedgecoe (2002), Hedgecoe (2001b), and Hedgecoe (2003) illustrates how the discourse surrounding conditions such as schizophrenia exemplifies enlightened geneticization, wherein genetic causality is foregrounded while non-genetic determinants are implicitly marginalized. In contrast, he describes the incorporation of genetic explanations into public health narratives around diabetes as a form of “geneticization by stealth,” a more gradual and understated integration of genetic framing. These distinctions underscore the evolution of geneticization from overtly deterministic models to more subtle, yet still hierarchical, representations of causality within biomedical discourse (Hedgecoe, 2002).

More recently, Dingel et al. (2019) have applied the concept of enlightened geneticization to the context of addiction, illustrating how individuals integrate genetic explanations into their personal narratives. Their study shows that individuals often adopt genetic framings of addiction to mitigate stigma, using notions of genetic predisposition as a way to legitimize their experiences and reduce feelings of personal culpability. This reframing allows for a more compassionate understanding of addiction, positioning it within a biomedical discourse that emphasizes inherited vulnerability rather than moral failure. The application of the enlightened geneticization thesis in this context reflects a broader shift toward integrated models of causation, where both genetic and non-genetic influences are acknowledged, albeit unevenly in shaping health and behavior. This case underscores the evolving societal discourse around identity, responsibility, and biomedical legitimacy in relation to addiction (Dingel et al., 2019).

#### 3.1.2 Biological citizenship

The term “biological citizenship” was first introduced by Petryna (2002) in her ethnographic work, *Life Exposed: Biological Citizens After Chernobyl*, where she examined how individuals in post-disaster contexts negotiated medical care, legal recognition, and state support through claims rooted in biological harm. The concept was later elaborated by Rose and Novas (2005) in the context of genetic testing, healthcare access, and patient advocacy, where they argued that increasing engagement with genetic knowledge is reshaping how individuals conceptualize identity, responsibility, and health-related rights. Rose (2001) contended that individuals are increasingly called upon to act as biological citizens, exercising agency in relation to their genetic risks and health trajectories. Unlike enlightened geneticization, which retains a strong emphasis on genetic causality despite its acknowledgment of complexity, biological citizenship offers a more pluralistic framework. It situates genetic knowledge within broader social, cultural, and environmental contexts, enabling individuals to weave genetic information into their narratives of identity and belonging without reducing them solely to their biological components (Sharon, 2014). This shift reflects a growing recognition of the interplay between biosocial agency and technoscientific knowledge in shaping contemporary subjectivities.

In the earlier mentioned study by Dingel et al. (2019) on understanding addiction genetics through family history, they identified elements of both enlightened geneticization and biological citizenship within participants’ narratives. While both frameworks were evident, the authors argued that Rose (2001) notion of biological citizenship offered a particularly compelling lens for understanding these dynamics, owing to its flexibility and attentiveness to the multifaceted nature of identity. Their findings suggest that individuals construct personal identities that reflect a blending of genetic and non-genetic influences, situating biological risk within a broader context of social experience, family history, and cultural expectations. This process involved not only interpreting genetic predispositions but also responding to normative pressures regarding the management of genetic risk, particularly in relation to socially stigmatized conditions such as addiction. Thus, the study highlights the interplay between biomedical knowledge and lived experience, and the nuanced ways individuals negotiate responsibility, identity, and agency (Dingel et al., 2019).

#### 3.1.3 Biosociality

The concept of biosociality, introduced by Rabinow (1997), refers to the ways in which emerging genetic technologies reshape traditional modes of social organization by fostering new forms of community and identity grounded in biological or genetic knowledge. Rather than social ties being based solely on cultural or familial norms, biosociality suggests that individuals may come to identify and affiliate with others based on shared genetic traits or risks. This framework has been critically examined by Rapp (1999) and Finkler (2000), who drew on ethnographic research to interrogate how DNA and genetic discourse are integrated into everyday life. Rapp (1999) argues that biomedicine employs specific discursive strategies that encourage individuals to internalize genetic categories and see themselves as part of emerging biosocial groups. Yet, these messages are not passively absorbed; they are filtered, resisted, or reshaped through various mediating influences, including religious beliefs, cultural traditions, social class, and ethnicity. Finkler (2000) critiques what she terms the medicalization of kinship, observing that biomedical narratives often privilege genetic relatedness over socially constructed forms of familial connection. In contrast to Rabinow’s vision of biosociality as generative of new, flexible kinship formations, Finkler contends that medical discourse may, in fact, constrain kinship definitions, reinforcing biological essentialism and marginalizing non-biological or chosen relationships, such as those established through marriage, adoption, or emotional bonds.

Biosociality is conceptualized as a framework that highlights the productive role of genetic markers in generating new social categories and affiliations (Arribas-Ayllon, 2016). Biosocial identities often emerge through the hybridization of traditional and novel identity categories, resulting in socially heterogeneous and context-dependent formations. Tsekeris and Alexias (2012) postulated that this concept illustrates the development of a biological sense of personal identity and social existence, enabling individuals to formulate genetic explanations of themselves and cultivate new relationships with figures of scientific and medical authority. Biosociality also gives rise to new modes of civic participation and activism, particularly among individuals affected by genetic conditions. These include efforts to challenge stigma and discrimination, advocate for improved access to medical information and healthcare services and mobilize for greater recognition of patient rights. In this way, biosociality is not merely a descriptive term but a lens through which to understand the dynamic interplay between genetics, identity, and collective action in contemporary biopolitical landscapes (Tsekeris and Alexias, 2012).

Biosociality, as articulated by Arribas-Ayllon (2016), extends well beyond the confines of the clinical setting, giving rise to new social assemblages that are formed outside traditional medical institutions. This challenges conventional notions of biomedicine as limited to anatomical depth and diagnostic authority, by emphasizing practices such as risk calculation, mutation identification, and genetic interpretation, which facilitate the creation of dynamic social networks. Genetic knowledge contributes to the formation of new subjectivities, as individuals and groups organize around shared genomic variants, constructing networks of associations that include categories, narratives, and expert-lay collaborations. In this context, patient organizations may coalesce around particular genetic conditions, providing not only access to medical expertise but also collective narratives, cultural traditions, and support structures that enable members to share experiences, advocate for intervention, and interpret their identities through a genetic lens (Arribas-Ayllon, 2016). Biosociality thus offers a robust framework for examining the evolving interplay between the state, scientific authority, community, and lay individuals, highlighting how genetic knowledge is productive of subjectivity and new forms of affiliation.

The rise of the Internet and digital health platforms has significantly shaped collective practices of genetic identification and knowledge exchange, enabling the formation of virtual communities centered around rare genetic conditions and specific mutations (Sharon, 2014). Sharon (2014) highlights the proliferation of websites, forums, and chat rooms that support nearly every known genetic disorder, offering patients, at-risk individuals, and caregivers spaces for information sharing, emotional support, and advocacy. These online communities serve as vital social and epistemic networks, where participants exchange personal experiences related to disease management, treatment navigation, and healthcare access. Moreover, they provide avenues for users to collaboratively interpret genetic risk, seek credible health information, and mobilize for policy or research advancements relevant to their conditions. Such interactions reflect the growing role of lay actors in co-producing genetic knowledge, thereby challenging traditional hierarchies of biomedical authority. As users gain scientific literacy and negotiate forms of distributed expertise, the boundary between experts, consumers, and producers becomes increasingly blurred (Sharon, 2014; Arribas-Ayllon, 2016). This shift not only empowers individuals but also reconfigures the relationship between patients and genetics professionals, positioning online communities as influential stakeholders in the broader genomic landscape.

### 3.2 Geneticization from a sociological dimension

#### 3.2.1 Implications for race

The role of Genetic Ancestry Tests (GATs) in the geneticization of race and identity has been critically examined in recent scholarship (Roth and Lyon, 2018; Hunt and Merolla, 2022; Strand and Källén, 2021). In particular, Strand and Källén (2021) explore this phenomenon through individuals who used GATs to assert Viking ancestry, illustrating how genetic data is not simply accepted at face value but is actively interpreted and integrated into personal narratives. Their study introduces the notion of “geneticized identities,” which emerge at the intersection of objective genetic findings and subjective meaning-making processes. Participants constructed diverse and often imaginative understandings of what it means to be a Viking, ranging from traits like warlikeness to entrepreneurial spirit demonstrating how GATs serve as a flexible platform for identity formation. Rather than reinforcing static or essentialist notions of ancestry, these interpretations underscore the symbolic and aspirational dimensions of genetic information in contemporary identity practices (Strand and Källén, 2021).

The identities constructed through GATs are deeply shaped by socio-historical narratives surrounding groups such as the Vikings, often drawing on culturally embedded associations with strength, exploration, and entrepreneurial prowess (Strand and Källén, 2021). Individuals frequently map these traits onto their genetic results, interpreting biological ancestry through the lens of popular and historical representations. As a result, attributes like restlessness, aggression, or business acumen are sometimes attributed to so-called “Viking genes,” effectively naturalizing cultural stereotypes by presenting them as inherent biological characteristics. This process reflects how geneticized identities are co-produced through both scientific data and cultural imagination. Importantly, the cultural and racial context plays a critical role in shaping these interpretations. Studies have shown that claims to Viking ancestry are often entangled with notions of whiteness and Nordic exceptionalism, serving as a vehicle through which individuals assert social belonging or symbolic status (Ahmed, 2007; Strand and Källén, 2021). In this sense, the appeal of certain ancestral narratives may not only reflect personal identity exploration but also reproduce socio-political hierarchies, reinforcing racialized and Eurocentric ideals within contemporary frameworks of genetic belonging.

These processes of identity construction illustrate a broader trend in which genetic information becomes a central lens for understanding the self. The prominence of commercial GATs encourages individuals to interpret their DNA as authoritative markers of identity, sometimes leading to the disavowal of previously held cultural affiliations (Jacoby, 2022). This shift contributes to a broader cultural narrative in which genetic markers are positioned as the definitive source of personal truth, effectively eclipsing lived experience, social context, and cultural heritage. Jacoby (2022) critiques this discourse for reinforcing a biologically deterministic model of identity, which reduces the richness of human experience to genetic data, obscuring the socio-cultural and political dimensions that fundamentally shape how identity is constructed and maintained. The geneticization of identity, she argues, signals a significant transformation in how individuals conceptualize the self, raising critical concerns about authenticity, belonging, and the implications of genetic essentialism in an era of increasing reliance on biomedical frameworks for personal meaning (Jacoby, 2022).

A growing body of scholarship has highlighted the role of GATs in prompting individuals, particularly white Americans to reconsider and, in some cases, reconfigure their racial self-identifications based on genetic data (Roth and Ivemark, 2018; Roth and Lyon, 2018; Hunt and Merolla, 2022). In their study, Hunt and Merolla (2022) found that a significant number of white participants reported modifying their racial identities upon learning of previously unknown ethnic affiliations revealed through GATs. This phenomenon signals a broader shift in which genetic information begins to supplant conventional markers of identity, such as cultural heritage and familial narratives. Commercial GAT enterprises contribute to this dynamic by framing race and ethnicity as primarily biological constructs, thereby reinforcing essentialist views that stand in contrast to sociological understandings of race as socially constructed. These companies often promote the notion that identity can be distilled to genetic composition alone, prioritizing biological determinism over experiential and cultural dimensions of selfhood. Within this framework emerges the concept of the “social deconstruction of whiteness,” wherein white individuals adopt alternative ethnic labels derived from their GAT results as a means of distancing themselves from the undifferentiated category of “white” (Hunt and Merolla, 2022). While this repositioning may allow for the exploration of more complex ancestral narratives, it also raises critical concerns. Specifically, such re-identification occurs within a sociopolitical landscape that continues to confer systemic advantages upon whiteness. As such, this development reflects a problematic outcome of the geneticization of race and identity, whereby biological narratives can obscure or even reinforce existing racial hierarchies.

Beyond white identity reconstruction, Peters (2023) recently examined how GATs shape the construction of racial and ethnic identities among Black individuals and families. Central to this analysis is the concept of “racial-genomic interest convergence,” which underscores the mutual dependence between the GAT industry and Black consumers. This convergence reflects how Black individuals, seeking reconnection with African ancestry disrupted by historical violence, become integral to an industry that simultaneously commodifies Black identities and asserts narrative authority over Blackness. Peters (2023) critiques the assumption that GATs offer comprehensive identity reconstruction, arguing instead that Black ancestry often remains “necessarily unfinished.” This sense of incompletion contributes to deeper frustrations around Black identity, introducing new questions and reinforcing ethno-racial boundaries (Peters, 2023). GATs tend to elevate genetic connections over cultural or communal ties, thus narrowing the framework through which identity can be understood beyond genomic data. Advertising by GAT companies frequently promotes DNA as a definitive marker of identity (Putman and Cole, 2020), a message that may conflict with the lived realities of Black families, who often value non-biological conceptions of kinship and belonging (Peters, 2023). These findings underscore the ongoing complexities of how race and identity are negotiated through genomic technologies amid the persistent legacies of racial injustice.

#### 3.2.2 Implications for ethnicity

Efforts to use genetic authority to define cultural and social identity have raised profound ethical and sociological concerns, particularly when applied to marginalized populations such as the Roma or the Romani people (Shmidt and Donohue, 2024). This is seen to be grounded in a sociobiological framework that assumes culture, behavior, and identity can be inferred from genetic traits, thereby advancing a racialist ideology that imposes static identities based on presumed biological differences (Cvorovic and Lynn, 2014). By this reductionist logic, stereotypes are reinforced and the political and social exclusion of minority groups are rationalized. Advocates of geneticization promote narratives that cast minorities as inherently deviant from dominant societal norms by interpreting cultural practices as genetically predetermined (Shmidt and Donohue, 2024). The resulting epistemic structure marginalizes more complex or contextual understandings of identity, favoring essentialist interpretations (Nguyen, 2020). Shmidt and Donohue (2024) contend that the fusion of sociobiology and race science continues to legitimize discriminatory practices against Roma communities, perpetuating structural inequality and stereotyping within both scholarly and public spheres. They call for a critical reassessment of these narratives, advocating for interdisciplinary engagement to disrupt the epistemic authority of geneticization and to challenge its detrimental impact on marginalized identities (Shmidt and Donohue, 2024).

The geneticization of ethnicity is also evident in anti-doping research, where biological determinism frequently overshadows social and cultural interpretation. Within this field, “ethnicity” is often conflated with race, allowing complex cultural identities to be reframed as biological variables (Hyun, 2017). This conflation facilitates interpretations of genetic variation as indicators of athletic potential or susceptibility to doping, rather than recognizing them within broader socio-cultural frameworks. A prominent example involves research on the UGT2B17 gene, which exhibits polymorphisms across ethnic groups. Findings from such studies have led to claims that certain ethnicities inherently possess biological traits affecting doping detectability, thereby positioning ethnicity as a genetic marker rather than a cultural identity (Hyun, 2017). Specifically, the deletion polymorphism of the UGT2B17 gene, found in approximately 66.7% of East Asians, has been framed in media discourse to suggest a genetic predisposition among Asian athletes to avoid detection, promoting the stereotype of East Asians as “born to cheat” (Hyun, 2017). This narrative is further complicated by associations between ethnic dietary habits and doping outcomes. For instance, compounds in green tea which is widely consumed in East Asia have been shown to inhibit UGT2B17 activity, potentially influencing testosterone levels and skewing testosterone/epitestosterone ratios. As a result, athletes from these backgrounds began altering culturally significant dietary practices to avoid suspicion, illustrating how genetic framing has pathologized cultural behaviors. Hyun (2017) argues that such interpretations reinforce essentialist assumptions by linking traditional diets to genetic predispositions for doping, ultimately framing ethnic identity through a narrow biological lens.

Media portrayals play a central role in perpetuating these simplifications. In particular, media portrayals of anti-doping studies frequently reduce complex findings to narratives that imply definitive racial or ethnic advantages, thereby reinforcing racial essentialism in the public imagination (Hyun, 2017). This distortion contributes to tangible consequences for athletes from specific ethnic backgrounds, fostering anxiety and prompting behavioral changes including reassessment of culturally significant dietary practices out of concern for how these might be perceived. Such representations obscure the nuanced interplay between culture and biology, instead advancing reductive views that equate ethnicity with genetic predisposition. In this context, the geneticization of ethnicity and diet within anti-doping science exemplifies how cultural identities are increasingly subsumed under frameworks of biological determinism.

#### 3.2.3 Implications for kinship and community

The use of genetic testing, particularly in contexts like ancestry research, allows individuals to explore their familial connections in new ways, which indicates that genetics does not merely inform individual identity but also redefines social relationships. For instance, the proliferation of GATs has enabled individuals to explore familial connections in novel ways, extending kinship networks to include distant or previously unknown relatives. This phenomenon gives rise to what some scholars describe as a new form of genealogical realism, wherein biological data redefines familial structures and expands the parameters of relatedness (Finkler, 2000; Franklin, 2013; Peters, 2023). Such developments reflect a contemporary iteration of biosociality, characterized by the construction of relationships grounded increasingly in genetic ties rather than purely social bonds (Arribas-Ayllon, 2016). In clinical contexts, genetics has also been shown to medicalize kinship, particularly through the use of family pedigrees to assess hereditary risk (Finkler, 2005). Nevertheless, scholars such as Rapp (1999) argue that pre-existing social definitions of kinship often override genetic framings, suggesting that socially grounded ties can displace genetically derived ones. Similarly, empirical studies by Lock (2007) and Weiner (2011) reveal that lay responses frequently diverge from clinical expectations—individuals may downplay or disregard genetic links that fail to resonate with their lived experiences or relational understandings. These findings collectively highlight the tensions between genetic and social conceptualizations of kinship in both medical and everyday contexts. See Table 3 for summary of findings on geneticization from a sociological dimension.

**Table 3:** Summary of findings—geneticization from a sociological dimension.

Subtheme	Key findings	Representative studies
Race	Genetic Ancestry Tests (GATs) are reshaping racial identity by promoting biologically grounded understandings of belonging. Users interpret DNA results through cultural narratives (e.g., “Viking genes”), reinforcing whiteness and Eurocentric ideals while reconfiguring racial self-identification.	Strand and Källén (2021), Hunt and Merolla (2022), Jacoby (2022) and Peters (2023)
Ethnicity	Genetic authority is used to biologically define cultural groups (e.g., Roma communities), perpetuating stereotypes and exclusion. In anti-doping science, ethnicity is conflated with biology, producing essentialist narratives that pathologize cultural practices and diets.	Shmidt and Donohue (2024) and Hyun (2017)
Kinship and community	Genetic testing reconfigures social relationships, creating new forms of “biosociality” based on shared genetic information. Genetic frameworks medicalize kinship, but social understandings often resist purely biological definitions of relatedness.	Franklin (2013), Weiner et al. (2017), Peters (2023), and Arribas-Ayllon (2016)

### 3.3 Geneticization from a bioethical dimension

Ethical engagement with the process of geneticization aims to develop comprehensive frameworks to guide the responsible innovation and application of genetic technologies (ten Have, 2012). These ethical frameworks are informed by distinct national, cultural, and legal contexts, each reflecting specific moral values and rights-based discourses. Ethicists serve a crucial role in interrogating the broader implications of geneticization, assessing its effects across individual, institutional, and societal domains, and establishing normative criteria for the ethical acceptability of genetic interventions. Due to the intimate and sensitive nature of genetic data, policy development concerning its collection, use, and disclosure necessitates rigorous ethical and legal scrutiny (Shi and Wu, 2017). Within this landscape, bioethics operates both as an ideological force and as a mechanism for legitimizing biotechnological advances. It simultaneously functions as an evaluative lens, applying normative principles derived from dominant bioethical discourses to emerging genetic practices (Arnason and Hjörleifsson, 2007). To remain relevant, this discourse must be continually recalibrated to account for the multifaceted impacts of geneticization on both individual lives and social structures.

Ethical discourse surrounding geneticization often foregrounds the principle of individual autonomy as a foundational value (ten Have, 2012). Yet, this emphasis is increasingly complicated by state-led interventions that prioritize collective concerns such as public health or national security sometimes at the expense of individual rights (Gostin, 2000). In these scenarios, ethical considerations shift toward a model of collective responsibility, where the stewardship of genetic information involves a broad array of stakeholders, including healthcare professionals, genetic researchers, policymakers, and the public. The accelerated dissemination of genetic data via both traditional and digital media intensifies the ethical obligation to protect personal genetic information and to address potential harms. These concerns extend beyond individuals to encompass social groups and communities, highlighting the broader societal dimensions of genetic privacy and data governance.

Genetic information carries implications that extend well beyond the individual, potentially impacting family members, communities, social institutions, and, in certain contexts, entire nations. It may also be of considerable interest to third-party stakeholders such as insurers, employers, and law enforcement agencies. To address the ethical complexities of geneticization, ten Have (2001) introduced a four-level analytical model, comprising conceptual, institutional, cultural, and philosophical dimensions. This framework provides a structured approach for examining the multifaceted ethical considerations associated with the production and use of genetic knowledge. Expanding upon this model, Hedgecoe (2001a) highlighted the critical role of interdisciplinary engagement particularly among social scientists and philosophers across the first three levels. Such collaboration is essential for generating contextually nuanced and ethically sound responses to the evolving challenges posed by geneticization.

The development of genetic technologies frequently centers on the ideals of personalized medicine and individual autonomy in health management, often at the expense of acknowledging the profound influence of environmental and social determinants of disease (Brothers and Rothstein, 2015; Santaló and Berdasco, 2022). In response, mainstream bioethics must expand its evaluative scope to engage more critically with the process of geneticization, incorporating frameworks capable of addressing the broader societal ramifications of these technologies. This entails not only assessing their downstream effects but also interrogating the upstream ideological assumptions that shape their inception, design, and implementation.

#### 3.3.1 Concerns regarding genetic discrimination and stigma

The discourses surrounding geneticization are embedded with substantial cultural and political implications. One key concern is the capacity of genetic information to shape social identities, raising ethical issues related to genetic discrimination and the potential reinforcement of racial and ethnic stereotypes. Genetic testing frequently employs ethno-racial classifications that risk conflating intricate social hierarchies with ostensibly objective genetic data. This conflation can influence individuals’ social positioning and affect how they navigate relationships within these socio-genetic frameworks (Jacoby, 2022).

The broader ethical concerns surrounding geneticization include its potential to reinforce existing stereotypes and biases, particularly against marginalized populations (Heredia et al., 2017). The risk of genetic discrimination where genetic data is misused to stigmatize individuals or groups contributes to the perpetuation of harmful narratives that shape and constrain identity formation (Domaradzki, 2019). As individuals increasingly interpret their identities through the lens of genetic information, there emerges a heightened vulnerability to internalized and social stigma, especially among those who are aware of a genetic predisposition to certain diseases.

The implications of geneticization become especially pronounced in institutional contexts, such as military recruitment, where the medicalization of performance through genetic and genomic assessments risks reducing complex human capabilities to predetermined genetic traits (Roberts, 2015). By emphasizing heredity over experience, training, and demonstrated competence, such practices echo historical eugenics discourses and reinforce social hierarchies, potentially valorizing biased models of aptitude along racial and gender lines (Roberts, 2015). These institutional applications underscore the urgent need to critically evaluate the ethical ramifications of embedding genetic determinism into frameworks that govern human evaluation and opportunity.

Ethical dilemmas also emerge in the context of genetic enhancements, which extend the logic of geneticization into the realm of human augmentation (Leźnicki, 2020). Distinctions between therapeutic interventions, aimed at treating disease, and non-therapeutic enhancements, intended to improve innate abilities, raise fundamental questions regarding the alteration of human nature. Bioethical debates often frame these concerns through contrasting perspectives: personalistic Christian ethics tend to reject modifications perceived as violating divine intent, while utilitarian approaches may support interventions that enhance well-being (Leźnicki, 2020). Echoing concerns from institutional applications of genetic data, the potential resurgence of eugenic thinking in enhancement practices could facilitate exclusion or marginalization of individuals with genetic impairments, further entrenching social inequality (Roberts, 2015; Leźnicki, 2020). Despite technological feasibility, resistance from bioethicists and restrictive legal frameworks continues to temper the advancement of enhancement-focused genetic research, highlighting the ongoing negotiation between scientific possibility and ethical responsibility.

#### 3.3.2 Geneticization of education

The emerging geneticization of education represents a paradigm shift in how educational abilities and outcomes are conceptualized—one that increasingly attributes such characteristics to genetic determinants. This perspective, as described by Matthews (2024), draws an analogy to the process of medicalization: just as diverse human conditions have been reframed within biomedical frameworks, educational traits such as intelligence, mathematical aptitude, and reading proficiency are now being interpreted through the lens of genetic influence. In this context, geneticization implies a growing tendency to regard these traits as primarily encoded in DNA, thereby diminishing the perceived significance of pedagogical strategies, learning environments, and socio-cultural factors in shaping educational achievement.

The geneticization of education has progressed significantly over recent decades, particularly with the rise of genomic research and the widespread use of genome-wide association studies (GWAS), which aim to identify specific genetic variants linked to educational outcomes such as mathematical ability, reading skills, and overall educational attainment (Rietveld et al., 2013; Lee et al., 2018). A pivotal shift occurred in the early 2000s, as researchers increasingly pursued genetic correlations of educational traits, marking a key turning point in the genetic framing of education. Matthews (2024) offers a critical perspective on this trend, emphasizing the limited predictive and explanatory power of these studies and raising concerns about the robustness of claims that position genetic factors as the primary determinants of educational achievement.

The geneticization of education carries significant implications that extend beyond the scientific sphere into bioethical considerations and broader societal consequences. Central concerns center on the potential for exacerbating educational inequalities and enabling discrimination grounded in perceived genetic predispositions. With the growing accessibility of DTC genetic testing, there is an increasing risk that parents and educators may develop biases based on assumptions about a child’s genetic potential. Matthews (2024) suggests such biases could shape parental expectations and educational strategies, thereby reinforcing a self-fulfilling prophecy in which children with lower genetic scores are afforded fewer opportunities and resources, entrenching existing patterns of disadvantage. See Table 4 for summary of findings on geneticization from a bioethical dimension.

**Table 4:** Summary of findings—geneticization from a bioethical dimension.

Subtheme	Key findings	Representative studies
Ethical frameworks	Bioethics provides tools for evaluating the moral implications of genetic technologies but must evolve to address collective and societal concerns beyond individual autonomy.	Sharon (2014) and Roberts (2015)
Discrimination and stigma	Genetic information risks reinforcing racial and social stereotypes, legitimizing discrimination in institutional settings (e.g., military recruitment) and through enhancement debates.	Jacoby (2022), Roberts (2015), Domaradzki (2019), and Leźnicki (2020)
Education	Educational attainment and ability are increasingly geneticized through GWAS studies, diminishing recognition of socio-cultural influences. This trend risks exacerbating inequality and bias in educational systems.	Matthews (2024)

### 3.4 Geneticization from a clinical dimension

The alignment between genotypic and phenotypic information frequently presents challenges, underscoring the inherent complexity of pathological classifications (Rabeharisoa and Bourret, 2009). This misalignment can generate psychological and social distress, largely stemming from the uncertainty surrounding disease probability. Nonetheless, genetic diagnosis may also empower individuals to pursue screening and preventive interventions, offering a means to potentially reduce the impact of genetic susceptibilities.

In this regard, the notion of genetic disease itself is increasingly contested. As virtually all diseases involve genetic components, the term risks losing specificity and meaning, potentially becoming universally applicable (Dekeuwer, 2017). This conceptual broadening raises what Dekeuwer (2017) terms the “causal selection problem”: the difficulty of determining which genetic factors should be regarded as primary contributors to disease, particularly in multifactorial conditions. This critique challenges simplistic models of genetic causation by foregrounding the intricate interplay between genetic and environmental factors. The concept of geneticization, often linked to deterministic and reductionist views of human life and behavior, may yield troubling social consequences such as the perception that genetic issues are best addressed through genetic selection, a view some interpret as implicitly eugenic (Dekeuwer, 2017).

The institutionalization of these perspectives within biomedical infrastructures further illustrates how geneticization becomes embedded in scientific practice. This trend is evident in the evolution from Mendelian Inheritance in Man (MIM) to Online Mendelian Inheritance in Man (OMIM), which reflects a broader shift toward molecular conceptualizations of disease. According to Amberger et al. (2015) and Ankeny (2017), OMIM has adopted stricter nosological criteria to navigate the tension between offering comprehensive genetic data and avoiding an excessively geneticized view of disease. Ankeny (2017) further argues that the institutionalization of MIM and OMIM as central tools in clinical genetics has entrenched a genetically centered framework for disease understanding and that the accessibility of these databases has amplified this geneticized perspective.

#### 3.4.1 Geneticization effects on specific diseases

The social construction of autism has undergone a significant transformation, shifting from its initial framing as a psychiatric disorder grounded in behavioral and developmental paradigms to its contemporary classification as a genetically determined condition (Melendro-Oliver, 2004; Nadesan, 2013). Navon and Eyal (2016) argue that the genetic framing of autism has fostered the emergence of biosocial communities among parents and children, enabling shared genetic understanding to serve as a foundation for emotional bonding and collective identity This reframing has helped parents move away from experiences of blame toward a sense of shared identity and advocacy, allowing them to view themselves as “experts on their own children.” In doing so, they have articulated subjective interpretations of autism that reinforce their roles within these communities, which seek both care and societal recognition (Navon and Eyal, 2016). The use of genetic terminology establishes a discursive framework that enables individuals to articulate, represent, and, in doing so, construct and solidify autism as a distinct biosocial entity. The adoption of genetic terminology has also established a discursive framework through which autism is articulated and socially constructed as a distinct biosocial entity (Silverman, 2011). As genetic classifications of autism have become more prominent, diagnostic criteria have expanded, contributing to its current status as a spectrum disorder. This expansion has increased genetic heterogeneity and introduced new complexities into autism identities, demonstrating that geneticization interacts dynamically with evolving social perceptions and the fluid nature of biosocial identity (Navon and Eyal, 2016).

#### 3.4.2 Geneticization of reproduction, genetic screening procedures, and medicine

The geneticization of reproduction has been characterized as a transformative shift in the application of Assisted Reproductive Technologies (ART), wherein the focus moves from addressing infertility to the active selection or engineering of embryos based on genetic traits (Alon et al., 2021). This shift carries significant ethical concerns, including the risk of fostering modern forms of eugenics, exacerbating social inequalities, and intensifying discrimination based on genetic characteristics (Asch and Barlevy, 2012; Alon et al., 2021). Within the context of reproductive genetic services (RGS), Alon et al. (2021) describe this process as encompassing the use of technologies such as Preimplantation Genetic Testing (PGT) to select embryos for specific traits, raising the prospect of human enhancement. This shift marks a transition from PGT’s initial focus on detecting severe, early-onset monogenic disorders to broader applications that include less severe, late-onset polygenic conditions, thereby expanding the scope and ethical complexity of genetic testing in reproduction (Klitzman et al., 2008; Batzer and Ravitsky, 2009). Alon et al. (2021) conclude that the geneticization of reproduction embodies a complex intersection of technological advancement, ethical considerations, and societal values, thereby posing substantial challenges for regulatory frameworks tasked with balancing individual reproductive autonomy against broader social consequences.

Closely related developments can be observed in the domain of prenatal diagnosis (PND), where the integration of genetic testing has fundamentally reshaped understandings of pregnancy and fetal health. The increasing association between PND and genetic evaluation, a process often described as the “geneticization of the unborn,” emerged during the 1960 and 1970s and transformed both medical practice and social perceptions of prenatal care (Löwy, 2014). While PND initially aimed to identify hereditary conditions within families, advancements in cytogenetics broadened its scope to include chromosomal anomalies such as those linked to Down syndrome (Löwy, 2014). The concept of “genetic abortion”- the termination of pregnancies based on the detection of fetal malformations gained prominence during this period, illustrating the expanding connection between PND and genetic frameworks, even in cases where conditions were not strictly genetic (Löwy, 2014).

A pivotal role was played in reinforcing the genetic framing of PND, by the emergence of genetic counselors as they became integral to guiding women through the complexities of prenatal testing, including deliberations regarding potential terminations (Stern, 2012). This development reflects the deepening integration of genetics into reproductive decision-making and underscores the ethical challenges inherent in the genetic discourse surrounding prenatal care. Consequently, the geneticization of PND represents more than an advancement in diagnostic capabilities; it marks a broader cultural transformation in how pregnancy, disability, and reproductive rights are understood and navigated (Löwy, 2014). This process of geneticization functioned to obscure the limitations of medical science in preventing or treating many congenital disorders, while simultaneously promoting an optimistic narrative centered on the promise of genetic science.

The social and institutional embedding of these technologies is further illustrated in the use of preimplantation genetic diagnosis and screening (PGD/PGS) within fertility clinics. In Spain, for instance, the implementation of such technologies has received broad support among healthcare professionals, though their adoption is deeply influenced by the interplay of regulatory, economic, and cultural factors (Pavone and Arias, 2012). These mediating factors contribute to the broader process of geneticization more than the scientific or technological aspects and therefore a need to scrutinize these socio-institutional factors is highlighted more than the application of the technologies in the process of geneticization (Pavone and Arias, 2012).

The process of geneticization is seen not to be confined only to reproductive medicine but extends into the broader clinical landscape. In addition to the “geneticization of the unborn,” Löwy (2022) argues that a parallel “geneticization of the clinics” has occurred, particularly within the evolving paradigms of oncology, where genomic analysis has become a routine component of cancer treatment. This shift reflects a convergence of research and clinical care, effectively blurring traditional boundaries between the two domains (Cambrosio et al., 2018). The incorporation of genetic data into clinical practice introduces new organizational dynamics, offering the potential for personalized medical interventions while simultaneously raising concerns about the cost, equity, and accessibility of such treatments (Jones, 2013).

At a deeper epistemological level, the expansion of precision medicine reflects not only a technical shift but also the rise of a new scientific ideology. Drawing on Rheinberger (2013) conception of scientific ideologies as systems of thought that transcend empirical boundaries, precision medicine can be seen as a modern manifestation of the genetic ideal—one that offers hyperbolic representations of biological control while remaining partially detached from clinical reality (Löwy, 2022). By aligning precision medicine with this tradition, Löwy (2022) situates it within a broader historiographical context, prompting critical reflection on its epistemological and cultural implications for the fields of biology, genetics, and medicine.

The reach of geneticization extends even further when viewed through the lens of traditional, complementary, and alternative medicine (TCAM). This has been explored by Shim and Kim (2020), who examined how genetic beliefs influence TCAM utilization across different national contexts. Their findings reveal considerable variation, with many countries outside East Asia exhibiting a strong negative correlation between genetic beliefs and the use of TCAM. Shim and Kim (2020) argue that these patterns are shaped significantly by institutional contexts, which mediate the relationship between belief systems and medical practices. In settings where TCAM is formally recognized as a legitimate and accessible component of healthcare, individuals who hold genetic beliefs are nonetheless more inclined to use TCAM (Shim and Kim, 2020). This indicates that geneticization is not solely a cognitive or conceptual transformation but is also contingent upon the broader medical and institutional structures within which individuals make health-related decisions. See Table 5 for summary of findings on geneticization from a clinical dimension.

**Table 5:** Summary of findings—geneticization from a clinical dimension.

Subtheme	Key findings	Representative studies
Disease classification	The definition of “genetic disease” has expanded, blurring distinctions between genetic and environmental causation. Databases like OMIM reinforce a genetically centered disease model.	Dekeuwer (2017) and Ankeny (2017)
Specific conditions (e.g., autism)	Autism’s framing has shifted from behavioral to genetic, fostering new biosocial communities and reshaping identity politics around neurodiversity.	Navon and Eyal (2016)
Reproduction and screening	Reproductive technologies increasingly focus on embryo selection and genetic screening, raising eugenic and social equity concerns. Geneticization extends to prenatal care and clinical practice.	Alon et al. 2021, Löwy (2022), and Pavone and Arias (2012)
Precision and alternative medicine	Precision medicine and TCAM illustrate how genetics penetrates diverse clinical paradigms, shaping both biomedical ideologies and complementary health practices.	Löwy (2022) and Shim and Kim (2020)

## 4 Discussion

This scoping review examined contemporary scholarly discourse on geneticization and assessed the extent to which Abby Lippman’s original critiques remain relevant in light of recent developments. The findings affirm that while the conceptual core of geneticization, i.e., the increasing attribution of health and identity to genetic factors persists, it has evolved into more nuanced and multidimensional frameworks across disciplines.

### 4.1 Geneticization: from determinism to nuance

Early critiques of geneticization, such as Lippman (1991) warning against reductionist and deterministic thinking remain foundational. However, recent studies suggest that contemporary discourse has moved beyond simplistic binaries. The works of Arribas-Ayllon (2016) and Weiner et al. (2017) have demonstrated that genetic explanations are now often embedded within more complex understandings that incorporate social, environmental, and psychological factors. The concept of enlightened geneticization exemplifies this shift, where genes remain central, yet are contextualized within broader causal networks (Hedgecoe, 2001b). This supports Weiner et al. (2017) conclusion that the original fears of total genetic determinism have only partially materialized, as modern accounts increasingly reflect dynamic, hybrid models of causation.

### 4.2 Social identity, race, and ethnicity

The review highlights that geneticization today extends far beyond clinical genetics into domains of social identity and race. The proliferation of GATs has played a critical role in reshaping how individuals understand and form their identities. While some users find empowerment in discovering genetic links to ancestral groups, others experience disorientation or loss of previously held cultural identities (Strand and Källén, 2021; Jacoby, 2022). This duality illustrates the productive yet potentially reductive nature of genetic information, which can both enrich and constrain identity narratives. Studies also indicate that geneticization can reinforce essentialist understandings of race, particularly when GAT results are interpreted as biologically determinative of cultural traits (Peters, 2023; Hunt and Merolla, 2022). The phenomenon of racial-genomic interest convergence and critiques of genetic reductionism in the case of Roma identity underscore the political and ethical stakes of geneticized identities in reinforcing existing hierarchies (Peters, 2023; Shmidt and Donohue, 2024).

The concepts of “biosociality,” “enlightened geneticization” and “biological citizenship” or “biocitizenship” highlight the growing significance of genetic and biological classifications in establishing community affiliations (Rabinow, 1997; Hedgecoe, 2001b; Rose, 2001). These frameworks illustrate how individuals increasingly identify and connect with particular groups based on shared genetic or biological traits, reflecting a broader transformation in the understanding of social belonging within contemporary society (Sharon, 2014). Such identities are rooted in the recognition and negotiation of genetic relationships, which facilitate new forms of community engagement and collective advocacy centered around biological identities. Moreover, these concepts facilitate the categorization of individuals or groups based on their patterns of interaction with genetic knowledge, allowing for a more context-sensitive and accurate characterization of their lived experiences and biomedical conditions.

### 4.3 Bioethical dimensions and ELSI

The ethical concerns first raised by Lippman remain highly relevant, particularly regarding autonomy, consent, and genetic discrimination. The review identifies a persistent gap between mainstream principlist bioethics and the sociocultural realities of geneticization, echoing critiques by ten Have (2001) and Arnason and Hjörleifsson (2007). As genetic knowledge becomes more accessible through media and DTC testing, ethical frameworks must evolve to address not only individual rights but also collective harms, particularly among historically marginalized populations (Roberts, 2015; Leźnicki, 2020; Shmidt and Donohue, 2024). The review also finds that stigma and selective disadvantage remain pressing issues. In areas like education and military recruitment, the integration of genetic frameworks has raised alarm over the potential to categorize individuals based on their presumed genetic potential (Roberts, 2015; Matthews, 2024). These trends reinforce the need for continuous ethical scrutiny and socially responsive policy development.

### 4.4 Clinical and reproductive contexts

In clinical and reproductive domains, the findings affirm that geneticization has become deeply institutionalized, particularly in areas such as the application of PND, PGT and precision oncology (Löwy, 2014; Alon et al., 2021; Cambrosio et al., 2018). As genomic tools become central to medical diagnostics and reproductive decision-making, the boundaries between medical care and technoscientific ideologies blur. While these tools enhance predictive capacities, they also contribute to the medicalization of reproduction and the geneticization of the unborn, raising ethical concerns about eugenics, informed consent, and social equity (Löwy, 2014). Moreover, developments such as OMIM exemplify how digital genomic repositories reinforce genetic framings of disease. As Ankeny (2017) notes, OMIM represents not just a shift in format but a transformation in how disease is conceptualized, placing genotype at the core of diagnosis and classification.

Previous studies have examined the extent to which conditions such as cystic fibrosis, mental illnesses, and breast cancer have undergone processes of geneticization. For instance, Phelan (2005) work on mental illnesses such as schizophrenia and depression highlights the increasing emphasis on genetic explanations, particularly through the lens of familial aggregation. This perspective is further supported by twin and adoption studies, which have consistently shown that genetic factors play a more significant role than non-genetic influences in the etiology of schizophrenia (Kendler and Diehl, 1993). Similar patterns are observed in autism research, with Navon and Eyal (2016) illustrating how genetic framings have come to dominate discussions of conditions previously not conceptualized in genetic terms. While this shift is not universally viewed as problematic, as Navon and Eyal (2016) acknowledge, concerns have been raised in specific contexts. In the case of breast cancer, for example, Sherwin and Simpson (1999) argue that the growing focus on genetic risk may marginalize environmental, nutritional, and broader social determinants of health, reflecting a key tension in the discourse on geneticization.

### 4.5 Cross-disciplinary tensions and global perspectives

A key finding emerging from this review is that the discourse on geneticization remains inherently cross-disciplinary yet fragmented, marked by epistemological tensions between the biological sciences, social sciences, and ethics. Sociologists and anthropologists focus on how genetic knowledge is shaped by social context and interpretation (Arribas-Ayllon, 2016; Weiner et al., 2017), while biomedical and clinical perspectives tend to see genetic information as an objective factor that determines health and identity (Ankeny, 2017; Löwy, 2022). This divergence creates a productive but uneasy dialogue: sociologists critique the reductionism of biomedical narratives, whereas clinicians often regard these critiques as obstructive to precision medicine’s progress. Similarly, within bioethics, debates over enhancement and eugenics reveal contradictions between utilitarian and deontological approaches (Leźnicki, 2020). The former often prioritize potential benefits of genetic optimization, while the latter caution against the moral risks of commodifying life. These disciplinary tensions illustrate the ongoing need for integrative frameworks that bridge empirical genetics and critical social inquiry, acknowledging both the epistemic authority of molecular science and the sociocultural contexts that shape its interpretation.

An enduring tension within biomedicine concerns the gap between the recognized importance of genetics in determining phenotypes and the limited realization of genomics’ early promises. Despite advances in sequencing and bioinformatics, the predictive and therapeutic power of genomics has not fully translated into clinical outcomes, largely due to the complex interactions between genes, environments, and lifestyles (Singhal et al., 2023; Brandes et al., 2022; Joyner and Prendergast, 2014). This gap has fueled growing interest in epigenetics, which positions gene expression as dynamic and environmentally responsive rather than fixed, thereby complicating the deterministic narratives underpinning geneticization (Landecker and Panofsky, 2013; Chellappoo et al., 2025). Yet even as epigenetics offers a more relational model of biology, it can inadvertently reinforce molecular reductionism by maintaining the primacy of genetic mechanisms over social and structural determinants of health (Meloni, 2015).

Comparatively, the dynamics of geneticization vary markedly between the Global North and Global South. In the Global North, particularly in North America and Western Europe, geneticization is tightly linked to consumer genomics, reproductive choice, and personalized medicine, framed within liberal narratives of autonomy and technological progress (Roth and Lyon, 2018; Alon et al., 2021). Conversely, in the Global South, geneticization often unfolds within contexts of constrained resources, limited regulatory capacity, and historical inequities in scientific infrastructure (Adebamowo et al., 2018; Jiwani et al., 2025). Here, genetic testing programs are frequently mediated by global health agendas or philanthropic interventions that may inadvertently reproduce forms of epistemic dependency. For instance, genomic initiatives in Africa and Latin America have raised concerns about data colonialism—the extraction of genetic data without equitable benefit-sharing or local governance mechanisms (Mulder et al., 2017; Helm et al., 2023; Adebamowo et al., 2018). Moreover, the local moral worlds of genetics differ: while Northern contexts tend to individualize risk, many Southern settings interpret genetic knowledge through collective or familial lenses (Helm et al., 2023). These disparities underscore that geneticization is not a universal trajectory but a situated process, reflecting global asymmetries in power, access, and knowledge production.

### 4.6 Policy implications

Geneticization raises urgent policy concerns across these critical domains:

- *Genetic privacy* demands urgent attention as genomic databases expand across public and private domains. International regulatory frameworks such as the EU’s GDPR and UNESCO’s Universal Declaration on the Human Genome and Human Rights offer partial protections but lack global enforceability. Stronger cross-border governance mechanisms are needed to prevent genetic surveillance, data exploitation, and discrimination in employment or insurance contexts (Shi and Wu, 2017).
- *Genetic education* must evolve beyond scientific literacy to include ethical and sociocultural literacy. Incorporating ELSI-focused curricula in medical and secondary education can empower individuals to interpret genetic information critically and resist deterministic narratives. Public engagement initiatives should be inclusive, addressing how race, class, and disability intersect with genomic knowledge and its social applications.
- *Reproductive rights* remain a contested arena in the age of geneticization. The expansion of prenatal and preimplantation genetic testing raises questions about informed consent, equity of access, and the potential normalization of selective reproduction (Löwy, 2014; Alon et al., 2021). Policymakers must ensure that reproductive autonomy is preserved without enabling coercive or discriminatory practices, particularly against those with disabilities or lower socioeconomic status. Ethical oversight bodies should prioritize transparency, equitable access, and counseling practices that foreground social context alongside genetic information.
- *Governance of commercial genomics and algorithmic bias* must become a central policy concern. As DTC genetic testing companies increasingly integrate AI-driven risk prediction and ancestry algorithms, there is a pressing need for transparency in how data are processed, categorized, and monetized (Ramanan et al., 2025). The opacity of proprietary algorithms risks reinforcing racialized classifications and amplifying biases embedded in genomic reference databases dominated by populations of European descent (Basu, 2023). Regulatory frameworks should therefore mandate algorithmic accountability, independent auditing of genomic databases, and equitable data representation from underrepresented populations to prevent the reproduction of systemic bias through genetic technologies.

### 4.7 Future research directions

Future research should aim to broaden the scope of inquiry both geographically and methodologically. There is a need for more empirical studies in underrepresented regions, particularly in the Global South, where genetic technologies may interact differently with cultural, legal, and socioeconomic structures. Additionally, cross-linguistic studies could help illuminate how geneticization discourses evolve in non-English academic and clinical contexts.

Given the evolving landscape of personalized medicine, genetic screening, and DTC testing, future work should explore real-world applications of genetic information and how individuals, communities, and professionals interpret and act on genetic data. In particular, longitudinal studies could better capture the dynamic nature of identity, kinship, and social stratification in the wake of geneticization.

Further, interdisciplinary research that bridges bioethics, sociology, STS and clinical practice is needed to critically evaluate the ethical and social implications of emerging genetic technologies. New frameworks should be developed to assess the intersectionality of geneticization, how it interacts with race, class, gender, and ability, and the impacts of algorithmic and AI-driven genomics in clinical settings.

Lastly, future work should continue to assess the relevance and evolution of Lippman’s original thesis in the context of postgenomic science, where complexity and interaction rather than reductionism now characterize much of genetic discourse. This would help determine whether geneticization remains a critical tool for conceptual critique or has transformed into a broader framework encompassing the biosocial reconfiguration of modern life.

### 4.8 Limitations of the study

While this review offers a comprehensive synthesis of contemporary literature on geneticization, several limitations should be acknowledged. First, the review was restricted to English-language, peer-reviewed publications from 2011 to 2024, potentially excluding relevant contributions published in other languages or formats, such as dissertations, conference proceedings, or gray literature. This language and publication bias may limit the global representativeness of the findings.

Second, despite rigorous screening, the conceptual ambiguity of geneticization across disciplines poses challenges in establishing consistent inclusion criteria. The term is used variably, sometimes centrally, other times peripherally, which may have led to the exclusion of studies that addressed relevant themes without using the exact terminology. Additionally, while backward citation tracking was employed, no forward citation analysis or systematic snowballing was conducted, which may have limited the identification of more recent or related works.

Third, although content analysis was conducted systematically, subjectivity in data interpretation cannot be fully eliminated. As with all qualitative reviews, thematic emphasis may reflect the analytical lens of the reviewers, despite efforts to enhance validity through author triangulation and iterative reading.

## 5 Conclusion

Recent scholarship reflects a conceptual shift in the use of geneticization—from an abstract noun denoting a broad phenomenon to an adjectival form employed to characterize specific domains increasingly framed in genetic terms. This linguistic and analytical transition is evident in reference to the geneticizing of race, identity, clinical practices, family structures, and education, among others. As a result, a substantial body of work over the past decade has concentrated on examining how diverse aspects of society and medicine have undergone processes of geneticization.

Although Abby Lippman’s original formulation of geneticization may now be viewed as conceptually outdated, the phenomenon it sought to capture remains highly relevant. With the continual advancement of genetic and genomic technologies and the growing body of knowledge linking specific DNA sequences to particular traits, the genetic framing of various aspects of life persists. Contemporary scholars continue to employ the term to denote the increasing attribution of genetic characteristics to social, medical, and individual phenomena.

Importantly, the findings reaffirm the utility of geneticization not merely as a descriptive term, but as a critical lens for monitoring the sociotechnical impact of genetic technologies. As the use of genetic information expands through practices like GAT, PND, PGT, and personalized medicine, geneticization provides a framework to evaluate whether such practices contribute to empowerment or marginalization. This is especially crucial in assessing the ELSI of the applications of these genetic and genomic technologies.

Looking forward, scholars and policymakers alike must engage proactively with the socio-political dimensions of genomic knowledge. This involves developing critical, context-sensitive frameworks that account for global inequalities in access, representation, and regulation. Policy responses should prioritize genetic privacy, equitable access to genomic healthcare, and inclusive public education that fosters genetic literacy without reinforcing determinism. In the sphere of reproductive rights, ethical oversight must balance autonomy with justice, ensuring that genetic technologies do not exacerbate structural discrimination or revive eugenic logics under new guises.

Ultimately, addressing the challenges of geneticization in the genomic era requires a sustained commitment to reflexivity, interdisciplinarity, and global equity. Scholars should continue interrogating how genetic knowledge shapes everyday life, while policymakers must ensure that governance frameworks uphold human dignity, social justice, and collective responsibility in the face of rapid biotechnological change.

## References

[ref1] Adebamowo S. N. Francis V. Tambo E. Diallo S. H. Landouré G. Nembaware V. . (2018). Implementation of genomics research in Africa: challenges and recommendations. Glob. Health Action 11:1419033. doi: 10.1080/16549716.2017.1419033, 29336236 PMC5769805

[ref2] Ahmed S. (2007). A phenomenology of whiteness. Fem. Theory 8, 149–168. doi: 10.1177/1464700107078139

[ref3] Ahmed Z. Zeeshan S. Lee D. (2023). Artificial intelligence for personalized and predictive genomics data analysis. Front. Genet. 14:1162869. doi: 10.3389/fgene.2023.1162869, 36936434 PMC10020608

[ref4] Alon I. Urbanos-Garrido R. Guimón J. (2021). Regulating reproductive genetic services: dealing with spiral-shaped processes and techno-scientific imaginaries. J. Assist. Reprod. Genet. 38, 305–317. doi: 10.1007/s10815-020-02017-9, 33405005 PMC7884509

[ref5] Amberger J. S. Bocchini C. A. Schiettecatte F. Scott A. F. Hamosh A. (2015). OMIM. Org: online Mendelian inheritance in man (OMIM®), an online catalog of human genes and genetic disorders. Nucleic Acids Res. 43, D789–D798. doi: 10.1093/nar/gku1205, 25428349 PMC4383985

[ref6] Ankeny R. A. (2017). Geneticization in MIM/OMIM®? Exploring historic and epistemic drivers of contemporary understandings of genetic disease. J. Med. Philos. 42, 367–384. doi: 10.1093/jmp/jhx013, 28641396

[ref7] Arnason V. Hjörleifsson S. (2007). Geneticization and bioethics: advancing debate and research. Med. Health Care Philos. 10, 417–431. doi: 10.1007/s11019-007-9088-9, 17705026

[ref8] Arribas-Ayllon M. (2016). After geneticization. Soc. Sci. Med. 159, 132–139. doi: 10.1016/j.socscimed.2016.05.011, 27191974

[ref9] Asch A. Barlevy D. (2012). “Disability and genetics: a disability critique of pre-natal testing and pre-implantation genetic diagnosis (PGD)” in eLS. Hoboken, New Jersey: Wiley-Blackwell.

[ref10] Barata L. P. Starks H. Kelley M. Kuszler P. Burke W. (2015). What DNA can and cannot say: perspectives of immigrant families about the use of genetic testing in immigration. Stanf. Law Policy Rev. 26:597, 26855553 PMC4743036

[ref11] Basu A. (2023). Use of race in clinical algorithms. Sci. Adv. 9:eadd2704. doi: 10.1126/sciadv.add2704, 37235647 PMC10219586

[ref12] Batzer F. R. Ravitsky V. (2009). “Preimplantation genetic diagnosis: ethical considerations” in The Penn Center guide to bioethics (New York: Springer), 339–354.

[ref13] Bener A. Al-Mulla M. Clarke A. (2019). Premarital screening and genetic counseling program: studies from an endogamous population. Int. J. Appl. Basic Med. Res. 9, 20–26. doi: 10.4103/ijabmr.IJABMR_42_18, 30820415 PMC6385533

[ref14] Brandes N. Weissbrod O. Linial M. (2022). Open problems in human trait genetics. Genome Biol. 23:131. doi: 10.1186/s13059-022-02697-9, 35725481 PMC9208223

[ref15] Brothers K. B. Rothstein M. A. (2015). Ethical, legal and social implications of incorporating personalized medicine into healthcare. Pers. Med. 12, 43–51. doi: 10.2217/pme.14.65, 25601880 PMC4296905

[ref16] Cambrosio A. Keating P. Vignola-Gagné E. Besle S. Bourret P. (2018). Extending experimentation: oncology’s fading boundary between research and care. New Genet. Soc. 37, 207–226. doi: 10.1080/14636778.2018.1487281

[ref17] Chellappoo A. Baedke J. Meloni M. (2025). From genetic to postgenomic determinisms: the role of the environment reconsidered: introduction to the collection ‘postgenomic determinisms: environmental narratives after the century of the gene’. Hist. Philos. Life Sci. 47:23. doi: 10.1007/s40656-025-00672-8, 40266445 PMC12018619

[ref18] Clarke A. E. Shim J. K. Mamo L. Fosket J. R. Fishman J. R. (2003). Biomedicalization: technoscientific transformations of health, illness, and US biomedicine. Am. Sociol. Rev. 68, 161–194. doi: 10.2307/1519765

[ref19] Cvorovic J. Lynn R. (2014). Intelligence and reproductive success of Bosniaks, Serbs and Roma in Serbia. Mank. Q. 54:434. doi: 10.46469/mq.2014.54.3.9

[ref20] Dara M. Dianatpour M. Azarpira N. Tanideh N. (2025). The transformative role of artificial intelligence in genomics: opportunities and challenges. Gene Rep. 41:102314. doi: 10.1016/j.genrep.2025.102314

[ref21] Dekeuwer C. (2017). “Conceptualization of genetic disease” in Handbook of the philosophy of medicine (Dordrecht, The Netherlands: Springer), 1–18.

[ref22] Dias R. Torkamani A. (2019). Artificial intelligence in clinical and genomic diagnostics. Genome Med. 11:70. doi: 10.1186/s13073-019-0689-8, 31744524 PMC6865045

[ref23] Dingel M. J. Ostergren J. Koenig B. A. McCormick J. (2019). “Why did I get that part of you?” understanding addiction genetics through family history. Public Underst. Sci. 28, 53–67. doi: 10.1177/0963662518785350, 29947292 PMC6342673

[ref24] Domaradzki J. (2019). Geneticization and biobanking. Pol. Sociol. Rev. 205, 103–118. doi: 10.26412/psr205.07

[ref25] Duster T. (2015). A post-genomic surprise. The molecular reinscription of race in science, law and medicine. Br. J. Sociol. 66, 1–27. doi: 10.1111/1468-4446.12118, 25789799

[ref26] Finkler K. (2000). Experiencing the new genetics: family and kinship on the medical frontier: University of Pennsylvania Press.

[ref27] Finkler K. (2005). Family, kinship, memory and temporality in the age of the new genetics. Soc. Sci. Med. 61, 1059–1071. doi: 10.1016/j.socscimed.2005.01.002, 15955406

[ref28] Flitner M. (2003). Genetic geographies. A historical comparison of agrarian modernization and eugenic thought in Germany, the Soviet Union, and the United States. Geoforum 34, 175–185. doi: 10.1016/S0016-7185(02)00090-8

[ref29] Franklin S. (2013). “From blood to genes?” in Blood and kinship: matter for metaphor from ancient Rome to the present (New York, NY: Berghahn), 285–320.

[ref30] Friedman A. Anderson T. L. (2024). Motivations for direct-to-consumer genetic testing: understanding interpretations of ancestry results. Qual. Sociol. 47, 543–569. doi: 10.1007/s11133-024-09569-7

[ref31] Gerhardt U. (1989). Ideas about illness: An intellectual and political history of medical sociology. Hampshire, England: Springer Nature Link.

[ref32] Gostin L. O. (2000). Public health regulation: a systematic evaluation. JAMA 283, 3118–3122. doi: 10.1001/jama.283.23.3118, 10865307

[ref33] Hedgecoe A. M. (2001a). Ethical boundary work: Geneticization, philosophy and the social sciences. Med. Health Care Philos. 4, 305–309. doi: 10.1023/A:1012075726550, 11760230

[ref34] Hedgecoe A. M. (2001b). Schizophrenia and the narrative of enlightened geneticization. Soc. Stud. Sci. 31, 875–911. doi: 10.1177/030631201031006004 11831294

[ref35] Hedgecoe A. M. (2002). Reinventing diabetes: classification, division and the geneticization of disease. New Genet. Soc. 21, 7–27. doi: 10.1080/14636770220122746

[ref36] Hedgecoe A. M. (2003). Expansion and uncertainty: cystic fibrosis, classification and genetics. Sociol. Health Illn. 25, 50–70. doi: 10.1111/1467-9566.t01-2-00324, 14498944

[ref37] Hedgecoe A. M. (2009). “Geneticization: debates and controversies” in eLS. Hoboken, New Jersey: Wiley-Blackwell.

[ref38] Helm P. de Götzen A. Cernuzzi L. Hume A. Diwakar S. Ruiz Correa S. . (2023). Diversity and neocolonialism in big data research: avoiding extractivism while struggling with paternalism. Big Data Soc. 10:20539517231206802. doi: 10.1177/20539517231206802

[ref39] Heredia N. I. Krasny S. Strong L. L. Von Hatten L. Nguyen L. Reininger B. M. . (2017). Community perceptions of biobanking participation: a qualitative study among Mexican-Americans in three Texas cities. Public Health Genomics 20, 46–57. doi: 10.1159/000452093, 27926908 PMC5453816

[ref40] Hoedemaekers R. ten Have H. (1998). Geneticization: the Cyprus paradigm. J. Med. Philos. 23, 274–287. doi: 10.1076/jmep.23.3.274.2585, 9736189

[ref41] Horstman K. (2008). “Lifestyle, genes and cholesterol: new struggles about responsibility and solidarity” in Genetics from laboratory to society: Societal learning as an alternative to regulation. (Hampshire, England: Springer), 64–89.

[ref42] Hunt W. Merolla D. M. (2022). Genes and race in the era of genetic ancestry testing: the geneticization of identity and the social deconstruction of whiteness. Sociol. Compass 16:e13011. doi: 10.1111/soc4.13011

[ref43] Hyun J. (2017). Geneticizing ethnicity and diet: anti-doping science and its social impact in the age of post-genomics. Front. Genet. 8:56. doi: 10.3389/fgene.2017.00056, 28536601 PMC5422433

[ref44] Jacoby K. (2022). Commercially geneticizing race, ethnicity, and nation: the implications of the discourse surrounding commercialized genetic tests on identity. Compass 1, 7–24.

[ref45] Jiwani T. Akinwumi A. Cheé-Santiago J. Egorova Y. Frassetto G. di Lazzaro Filho R. . (2025). Conceptualizing the public good for genomics in the global south: a cross-disciplinary roundtable dialogue. Front. Genet. 16:1523396. doi: 10.3389/fgene.2025.1523396, 40672394 PMC12264638

[ref46] Jones D. (2013). “The prospects of personalized medicine” in Genetic explanations (United States of America: Harvard University Press), 147–170.

[ref47] Joyner M. J. Prendergast F. G. (2014). Chasing Mendel: five questions for personalized medicine. J. Physiol. 592, 2381–2388. doi: 10.1113/jphysiol.2014.272336, 24882820 PMC4048096

[ref48] Kendler K. S. Diehl S. R. (1993). The genetics of schizophrenia: a current, genetic-epidemiologic perspective. Schizophr. Bull. 19, 261–285. doi: 10.1093/schbul/19.2.261, 8322035

[ref49] Klitzman R. Appelbaum P. S. Chung W. Sauer M. (2008). Anticipating issues related to increasing preimplantation genetic diagnosis use: a research agenda. Reprod. Biomed. Online 17, 33–42. doi: 10.1016/S1472-6483(10)60188-5, 18644221

[ref50] Kovanda A. Zimani A. N. Peterlin B. (2021). How to design a national genomic project—a systematic review of active projects. Hum. Genomics 15:20. doi: 10.1186/s40246-021-00315-6, 33761998 PMC7988644

[ref51] Landecker H. Panofsky A. (2013). From social structure to gene regulation, and back: a critical introduction to environmental epigenetics for sociology. Annu. Rev. Sociol. 39, 333–357. doi: 10.1146/annurev-soc-071312-145707

[ref52] Lee J. J. Wedow R. Okbay A. Kong E. Maghzian O. Zacher M. . (2018). Gene discovery and polygenic prediction from a genome-wide association study of educational attainment in 1.1 million individuals. Nat. Genet. 50, 1112–1121. doi: 10.1038/s41588-018-0147-3, 30038396 PMC6393768

[ref53] Leźnicki M. (2020). Bioethical aspects of human geneticization. Stud. Ecol. Bioeth. 18, 7–17. doi: 10.21697/seb.2020.18.3.02

[ref54] Lippman A. (1991). Prenatal genetic testing and screening: constructing needs and reinforcing inequities. Am. J. Law Med. 17, 15–50. doi: 10.1017/S0098858800007917, 1877608

[ref55] Lippman A. (1992). Led (astray) by genetic maps: the cartography of the human genome and health care. Soc. Sci. Med. 35, 1469–1476. doi: 10.1016/0277-9536(92)90049-V, 1485194

[ref56] Lippman A. (1993). Prenatal genetic testing and geneticization: mother matters for all. Fetal Diagn. Ther. 8, 175–188. doi: 10.1159/000263886, 8512644

[ref57] Lippman A. (1998). “The politics of health: Geneticization versus health promotion” in The politics of women’s health: Exploring agency and autonomy (Philadelphia: Temple University Press), 64–82.

[ref58] Lock M. (2007). “Biosociality and susceptibility genes: a cautionary tale” in Biosocialities, genetics and the social sciences (London: Routledge), 66–88.

[ref59] Lock M. (2015). Comprehending the body in the era of the epigenome. Curr. Anthropol. 56, 151–177. doi: 10.1086/680350

[ref60] Löwy I. (2014). How genetics came to the unborn: 1960–2000. Stud. Hist. Phil. Biol. Biomed. Sci. 47, 154–162. doi: 10.1016/j.shpsc.2014.05.015, 24968964

[ref61] Löwy I. (2022). Precision medicine: historiography of life sciences and the geneticization of the clinics. Ber. Wiss. 45, 487–498. doi: 10.1002/bewi.202200023, 36086833 PMC9545106

[ref62] Majumder M. A. Guerrini C. J. McGuire A. L. (2021). Direct-to-consumer genetic testing: value and risk. Annu. Rev. Med. 72, 151–166. doi: 10.1146/annurev-med-070119-114727, 32735764

[ref63] Mannette R. (2021). Navigating a world of genes: a conceptual analysis of gene fetishism, geneticization, genetic exceptionalism and genetic essentialism. Eur. J. Med. Genet. 64:104232. doi: 10.1016/j.ejmg.2021.104232, 33974995

[ref64] Matthews L. J. (2024). The geneticization of education and its bioethical implications. Camb. Q. Healthc. Ethics, 1–17. doi: 10.1017/S096318012400046X PMC12226822 39506329

[ref65] Melendro-Oliver S. (2004). Shifting concepts of genetic disease. Sci. Technol. Stud. 17, 20–33. doi: 10.23987/sts.55170

[ref66] Meloni M. (2015). Epigenetics for the social sciences: justice, embodiment, and inheritance in the postgenomic age. New Genet. Soc. 34, 125–151. doi: 10.1080/14636778.2015.1034850

[ref67] Mulder N. Adebamowo C. A. Adebamowo S. N. Adebayo O. Adeleye O. Alibi M. . (2017). Genomic research data generation, analysis and sharing–challenges in the African setting. Data Sci. J. 16:49. doi: 10.5334/dsj-2017-049

[ref68] Nadesan M. H. (2013). Constructing autism: unravelling the 'truth' and understanding the social. Cornwall, Great Britain: Routledge.

[ref69] Navon D. Eyal G. (2016). Looping genomes: diagnostic change and the genetic makeup of the autism population. Am. J. Sociol. 121, 1416–1471. doi: 10.1086/684201, 27092389

[ref70] Nguyen C. T. (2020). Echo chambers and epistemic bubbles. Episteme 17, 141–161. doi: 10.1017/epi.2018.32

[ref71] Pavone V. Arias F. (2012). Beyond the geneticization thesis: the political economy of PGD/PGS in Spain. Sci. Technol. Hum. Values 37, 235–261. doi: 10.1177/0162243911411195

[ref72] Peters C. (2023). Racial-genomic interest convergence and the geneticization of black families. J. Fam. Commun. 23, 294–309. doi: 10.1080/15267431.2023.2228315, 40837123 PMC12362720

[ref73] Petryna A. (2002). Life exposed: Biological citizens after Chernobyl. Princeton, New Jersey: Princeton University Press.

[ref74] Phelan J. C. (2005). Geneticization of deviant behavior and consequences for stigma: the case of mental illness. J. Health Soc. Behav. 46, 307–322. doi: 10.1177/002214650504600401, 16433278

[ref75] Putman A. L. Cole K. L. (2020). All hail DNA: the constitutive rhetoric of AncestryDNA™ advertising. Crit. Stud. Media Commun. 37, 207–220. doi: 10.1080/15295036.2020.1767796

[ref76] Rabeharisoa V. Bourret P. (2009). Staging and weighting evidence in biomedicine: comparing clinical practices in cancer genetics and psychiatric genetics. Soc. Stud. Sci. 39, 691–715. doi: 10.1177/0306312709103501

[ref77] Rabinow P. (1997). Essays on the anthropology of reason. Princeton, New Jersey: Princeton University Press.

[ref78] Ramanan V. Vinod R. Williams C. Ramachandran S. Venkatasubramanian S. (2025, 2025). “Principles and policy recommendations for comprehensive genetic DataGovernance” in Eighth AAAI/ACM conference on AI, ethics, and society (AIES 2025). Palo Alto, California: The Association for the Advancement of Artificial Intelligence. 10.1609/aies.v8i3.36701 PMC13077651 41988211

[ref79] Rapp R. (1999). Testing women, testing the fetus: the social impact of amniocentesis in America. New York: Psychology Press.

[ref80] Rheinberger H. (2013). Heredity in the twentieth century: some epistemological considerations. Public Cult. 25, 477–493. doi: 10.1215/08992363-2144616

[ref81] Rietveld C. A. Medland S. E. Derringer J. Yang J. Esko T. Martin N. W. . (2013). GWAS of 126,559 individuals identifies genetic variants associated with educational attainment. Science 340, 1467–1471. doi: 10.1126/science.1235488, 23722424 PMC3751588

[ref82] Roberts J. L. (2015). ‘Good soldiers are made, not born’: the dangers of medicalizing ability in the military use of genetics. J. Law Biosci. 2, 92–98. doi: 10.1093/jlb/lsv007, 27774184 PMC5033551

[ref83] Rose N. (2001). The politics of life itself. Theory Cult. Soc. 18, 1–30. doi: 10.1177/02632760122052020

[ref84] Rose N. Novas C. (2005). “Biological citizenship” in Global assemblages: technology, politics, and ethics as anthropological problems. (Oxford, UK: Blackwell Publishing), 439–463.

[ref85] Roth W. D. Ivemark B. (2018). Genetic options: the impact of genetic ancestry testing on consumers’ racial and ethnic identities. Am. J. Sociol. 124, 150–184. doi: 10.1086/697487

[ref86] Roth W. D. Lyon K. (2018). “Genetic ancestry tests and race: who takes them, why, and how do they affect racial identities” in Reconsidering race: social science perspectives on racial categories in the age of genomics, vol. 1, (New York, United States of America: Oxford University Press), 33–169.

[ref87] Santaló J. Berdasco M. (2022). Ethical implications of epigenetics in the era of personalized medicine. Clin. Epigenetics 14:44. doi: 10.1186/s13148-022-01263-1, 35337378 PMC8953972

[ref88] Sessa F. Chisari M. Esposito M. Karaboue M. A. A. Salerno M. Cocimano G. (2024). Ethical, legal and social implications (ELSI) regarding forensic genetic investigations (FGIs). J. Acad. Ethics 23, 617–637. doi: 10.1007/s10805-024-09582-z

[ref89] Sharon T. (2014). “New modes of ethical selfhood: Geneticization and genetically responsible subjectivity” in Human nature in an age of biotechnology: the case for mediated posthumanism (Dordrecht: Springer), 199–237.

[ref90] Sherwin S. Simpson C. (1999). “Ethical questions in the pursuit of genetic information: Geneticization and BRCA1” in Genetic information: acquisition, access, and control. (New York: Springer), 121–128.

[ref91] Shi X. Wu X. (2017). An overview of human genetic privacy. Ann. N. Y. Acad. Sci. 1387, 61–72. doi: 10.1111/nyas.13211, 27626905 PMC5697154

[ref92] Shim J. Kim J. (2020). Contextualizing geneticization and medical pluralism: how variable institutionalization of traditional, complementary, and alternative medicine (TCAM) conditions effects of genetic beliefs on utilization. Soc. Sci. Med. 267:113349. doi: 10.1016/j.socscimed.2020.113349, 33008647

[ref93] Shmidt V. Donohue C. R. (2024). Invincible racism? The misuse of genetically informed arguments against Roma in central and Eastern Europe. Romani Stud. 34, 111–134. doi: 10.3828/rost.2024.6

[ref94] Silverman C. (2011). Understanding autism: parents, doctors, and the history of a disorder: Princeton University Press.

[ref95] Singhal P. Verma S. S. Ritchie M. D. (2023). Gene interactions in human disease studies—evidence is mounting. Annu. Rev. Biomed. Data Sci. 6, 377–395. doi: 10.1146/annurev-biodatasci-102022-120818, 37196359

[ref96] Stempsey W. E. (2006). The geneticization of diagnostics. Med. Health Care Philos. 9, 193–200. doi: 10.1007/s11019-005-5292-7, 16850199

[ref97] Stern A. M. (2012). Telling genes: the story of genetic counseling in America. Baltimore, Maryland: JHU Press.

[ref98] Strand D. Källén A. (2021). I am a Viking! DNA, popular culture and the construction of geneticized identity. New Genet. Soc. 40, 520–540. doi: 10.1080/14636778.2020.1868988

[ref99] ten Have H. A. (2001). Genetics and culture: the geneticization thesis. Med. Health Care Philos. 4, 295–304. doi: 10.1023/a:1012090810798, 11760229

[ref100] ten Have H. A. (2012). “Geneticization: concept” in eLS. Hoboken, New Jersey: Wiley-Blackwell.

[ref101] Tricco A. C. Lillie E. Zarin W. O'Brien K. K. Colquhoun H. Levac D. . (2018). PRISMA extension for scoping reviews (PRISMA-ScR): checklist and explanation. Ann. Intern. Med. 169, 467–473. doi: 10.7326/M18-0850, 30178033

[ref102] Tsekeris C. Alexias G. (2012). Science, genetic knowledge and the human body. PRO 81, 67–78.

[ref103] Weiner K. (2011). Exploring genetic responsibility for the self, family and kin in the case of hereditary raised cholesterol. Soc. Sci. Med. 72, 1760–1767. doi: 10.1016/j.socscimed.2010.03.053, 20627500

[ref104] Weiner K. Martin P. Richards M. Tutton R. (2017). Have we seen the geneticisation of society? Expectations and evidence. Sociol. Health Illn. 39, 989–1004. doi: 10.1111/1467-9566.12551, 28271518

